# MiRNA-mediated EMT and CSCs in cancer chemoresistance

**DOI:** 10.1186/s40164-021-00206-5

**Published:** 2021-02-12

**Authors:** Bing Dong, Shiyu Li, Shuangli Zhu, Ming Yi, Suxia Luo, Kongming Wu

**Affiliations:** 1 Department of Molecular Pathology, The Affiliated Cancer Hospital of Zhengzhou University & Henan Cancer Hospital, Zhengzhou, 450008 China; 2 Department of Oncology, Tongji Hospital of Tongji Medical College, Huazhong University of Science and Technology, Wuhan, 430030 China; 3 Department of Medical Oncology, The Affiliated Cancer Hospital of Zhengzhou University & Henan Cancer Hospital, Zhengzhou, 450008 China

**Keywords:** miRNA, EMT, CSCs, Chemoresistance

## Abstract

Cancer stem cells (CSCs) are a small group of cancer cells, which contribute to tumorigenesis and cancer progression. Cancer cells undergoing epithelial-to-mesenchymal transition (EMT) acquire the chemoresistant ability, which is regarded as an important feature of CSCs. Thus, there emerges an opinion that the generation of CSCs is considered to be driven by EMT. In this complex process, microRNAs (miRNAs) are found to play a key role. In order to overcome the drug resistance, inhibiting EMT as well as CSCs phenotype seem feasible. Thereinto, regulating the EMT- or CSCs-associated miRNAs is a crucial approach. Herein, we conduct this review to elaborate on the complicated interplay between EMT and CSCs in cancer chemoresistance, which is modulated by miRNAs. In addition, we elucidate the therapeutic strategy to overcome drug resistance through targeting EMT and CSCs.

## Introduction

Cancer stem cells (CSCs) are a special subset of cancer cells, which have the ability to self-renew and contribute to tumor initiation, metastasis, and chemoresistance [1, 2]. So far, several surface markers of CSCs have been identified, such as CD24, CD44, CD133, and EpCAM, which facilitate the CSCs isolation and targeting [3, 4]. Importantly, aldehyde dehydrogenase (ALDH), ATP-binding cassette subfamily G member 2 (ABCG2), and c-kit have been additionally regarded as the CSC hallmarks, which contribute to chemoresistance by regulating drug metabolism or affecting the gene expression of drug efflux [5]. Recent studies have revealed that the generation of CSCs is likely driven by the epithelial-to-mesenchymal transition (EMT). EMT is a morphogenetic process, in which cancer cells lose their epithelial properties, such as the apical-basal polarity and cell junctions, while acquiring mesenchymal characteristics, including the increased capacity of migration and invasion[6]. Additionally, the activation of EMT confers the tumor cells with the capacity to resist various chemotherapeutics, which is also a crucial feature of CSCs [7].

MicroRNAs (miRNAs) are a part of non-coding single-stranded small RNAs (18–22 nucleotides) that can suppress gene expression through binding the 3’-UTR of target mRNA [8]. With the further understanding of miRNAs, researchers find miRNAs can function as oncogenes or tumor suppressors to modulate tumor cell proliferation, apoptosis, immune response, and reshape microenvronment [9–12]. Recently, a growing number of studies report miRNAs play a pivotal role in regulating the EMT program and the CSCs genesis [13]. However, we still have few insights into the complicated relationship between cancer chemoresistance and miRNA-mediated CSCs and EMT. Therefore, we conduct this review to elaborate on the mechanistic link between CSCs as well as EMT, and summarize the role of EMT- or CSCs-associated miRNAs in cancer chemoresistance.

## EMT program and CSCs

Cancer cells undergoing the EMT process express more mesenchymal markers including N-cadherin as well as vimentin, and diminish the epithelial markers expressions like E-cadherin [14]. Loss of E-cadherin has been recognized to be related to cancer metastasis and the poor prognosis since nearly 20 years ago, then how to activate EMT draws considerable attention [15]. Numerous studies have reported the intricate EMT-associated signal pathways, in which the TGF-β-SMAD signal pathway has been well accepted [16]. Furthermore, Wnt signaling also contributes to the activation of EMT [17]. However, the complex signal pathways primarily activate a relatively small group of transcription factors to induce the EMT process. These transcription factors are termed as EMT-inducing transcription factors (EMT-TFs), which are typically categorized into three different protein families, including the ZEB (ZEB1 and ZEB2), Snail (SNAI1 and SNAI2, also termed as Snail and Slug), and basic helix–loop–helix (Twist1 and Twist2) families [18]. Notably, the EMT-TFs usually regulate the expression of one another and act together to activate EMT. For instance, Snail can increase the expression of Slug, Twist1, and ZEB1, which is regarded as an upstream regulator [19]. Due to the reciprocal interactions, it is difficult to determine the exact function of individual EMT-TF.

Apart from being modulated by specific signal pathways, EMT-TFs are also controlled by other regulators, especially miRNAs. MiRNAs affect the EMT process by directly or indirectly regulating specific EMT-TFs. The well-known examples are miR-200 and miR-34 families, which downregulates the expression of Snail and ZEB, respectively [20, 21]. Though the mechanism of the activation is becoming gradually clear, there remain some key problems to solve. For example, the experimental and clinical observations suggest the EMT program is reversible and dynamic, but how cancer cells harboring mesenchymal properties become epithelioid again in the metastatic sites needs to be further explored.

CSCs are a small subpopulation of cancer cells with stem cell-like characteristics, including quiescence, self-renew, and slow cell cycle. A growing number of studies indicate conventional chemotherapeutics mainly target the bulk non-CSCs population instead of the rare CSCs population that indeed cause the clinical relapse [22]. Mechanism explorations reveal that CSCs are resistant to chemotherapy owing to their quiescent state, increased drug efflux, and activate DNA repair [23]. When the chemotherapy ceases, CSCs that escape from cytotoxicity will revive from quiescence and promote tumorigenesis. Thus, eradicating CSCs are becoming a promising therapeutic approach to overcome chemoresistance and achieve clinical cure. Currently, the identification of these subpopulations primarily depends on the high expression of ALDH and surface markers like CD44 and CD133 [24]. However, it is still urgent to find other potent CSCs markers to select the patients who likely resist drugs in the clinic due to inter-patient variations and tumor heterogeneity [25]. This is critical to precisely targeting CSCs without impairing those stem cells from normal tissues.

## The relationship between EMT and CSCs

**Fig. 1:**
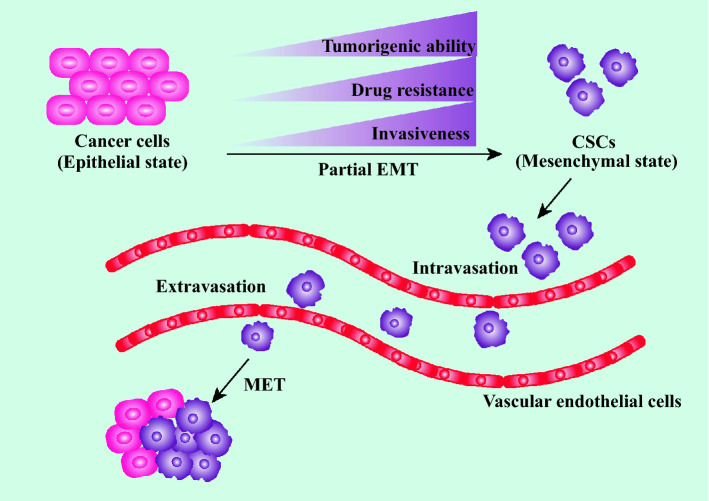
The connection between EMT and CSCs. CSCs can be generated by cancer cells that undergo a partial EMT process. Compared with cancer cells, CSCs are more invasive and drug-resistant and have great ability of tumor-initiation. CSCs are prone to intravasate into adjacent blood vessels. After entering the circulation, the metastatic CSCs will undergo transendothelial migration to extravasate into a new secondary site. Subsequently, CSCs can be transformed into cancer cells again via the MET process

Cancer cells undergoing EMT possess lots of stem-like traits, such as the elevated expression of CD44 and the increased capacity to form spheres [26]. These phenomena suggest EMT is closely related to the generation and maintenance of CSCs. Compelling evidence shows that CSCs may occur from progenitor cells or normal stem cells owing to the genetic and epigenetic mutations [27]. For instance, the overexpression of yes associated protein 1 (YAP1) that contributes to EMT can transform differentiated cancer cells into CSCs [28]. This characteristic example demonstrates that the abnormal expressions of EMT-related genes facilitate the generation of CSCs. CSCs live in a dynamic microenvironment, called niche, which is composed of stromal cells, immune cells, various cytokines and growth factors [29]. CSCs in such a niche are able to maintain their stemness state [30]. On one hand, the niche with hypoxia and high vascular intensity can directly maintain CSCs plasticity and survival [31]. On the other hand, the maintenance of CSCs can be reinforced by the EMT process under such a favorable microenvironment. For example, nestin is another CSCs marker that is upregulated by hypoxia-induced TGF-β-SMAD4 pathway activation [32]. Furthermore, cancer-associated fibroblasts and tumor-associated macrophages in the CSCs niche can secrete TGF-β to promote EMT, subsequently maintaining the CSCs features [33]. Additionally, CSCs have the potential to differentiate into non-CSCs. It is plausible that the aforementioned dynamic and reversibility of EMT can be partially attributed to the differentiation capacity of CSCs (Fig. 1).

The association between EMT and CSCs is supported by substantial experimental evidence concerning the mechanistic link. It is reported the activation of EMT and CSCs share similar signaling pathways, such as Wnt and Notch signals [34]. Notably, Scheel et al. found the autocrine of TGF-β and Wnt signal pathways of cancer cells were responsible for maintaining the EMT establishment and CSCs state. Blocking these autocrine signals could prevent cancer cells from acquiring CSCs properties even though the EMT program was activated [35]. Furthermore, Snail could facilitate the acquisition of dedifferentiated phenotype ultimately promoting the tumor-initiating capability by deacetylating active p53 [36]. Although substantial studies demonstrate the close relationship between EMT and CSCs, whether the EMT program is necessary for driving the CSCs phenotype remains to be further explored. Having insight into this problem may help us make a therapeutic decision in targeting mesenchymal cells or only precisely eradicating the small subpopulation of CSCs in the future.

## MiRNA-mediated EMT and CSCs in chemotherapy resistance

**Fig. 2:**
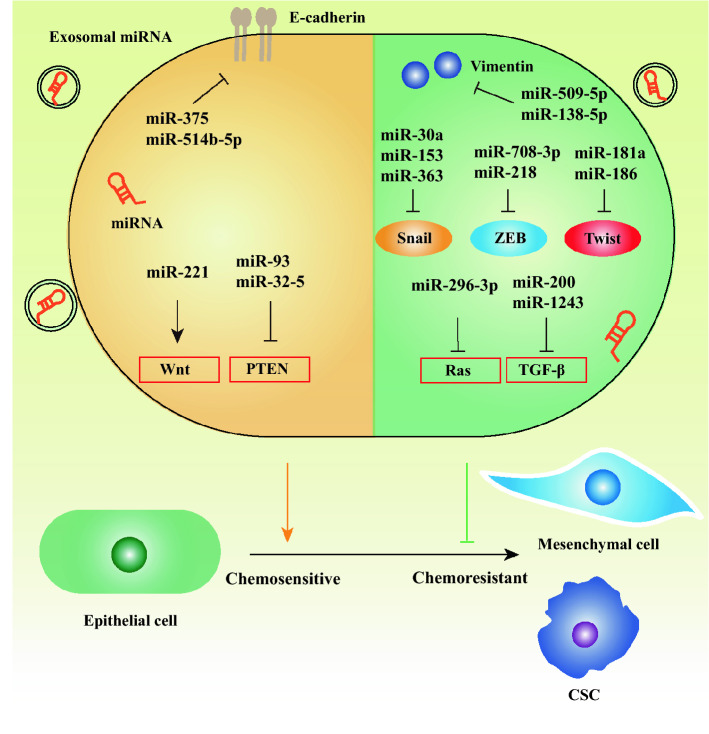
The mechanism by which some representative miRNA mediates EMT to affect chemotherapy resistance. MiRNA can regulate the EMT process by directly targeting the key proteins (E-cadherin and Vimentin) of EMT, modulating the expression of EMT-TFs (Snail, ZEB, and Twist), and influencing the EMT-associated signal pathways like Wnt, PTEN, TGF-β, and Ras signal. Furthermore, miRNA can be transferred by exosomes, which plays a key role in the EMT process. Cancer cells undergoing the EMT program exhibit the chemoresistant phenotype. In addition, the generation of chemoresistant CSC is partially attributed to the EMT

Though the studies about EMT focused on cancer metastasis at first, the link between EMT and cancer drug resistance has been increasingly recognized. Before the relation between EMT and CSCs was established, the mechanism of EMT-mediated drug resistance was unclear. In recent years, miRNAs as a hot topic have drawn considerable attention among numerous researchers. An increasing number of studies show miRNAs play a pivotal role in chemotherapy resistance, which is correlated to EMT or CSCs [37, 38]. Herein, we summarize the various roles of miRNAs in mediating EMT- or CSCs-associated chemoresistance (Fig. 2) (Table 1).

**Table 1:** The role of miRNA-mediated EMT and CSCs in drug resistance

miRNA	Cancer type	Chemotherapy	Role in chemosensitivity	Target	Ref.
miR-375	CC	Paclitaxel	Decrease	E-cadherin	[39]
miR-514b-5p	CRC	Cisplatin and irinotecan	Decrease	CDH1, CLDN1	[40]
miR-514b-3p	CRC	Cisplatin and irinotecan	Increase	FZD4, NTN1	[40]
miR-125b	CRC	5-FU	Decrease	APC	[42]
miR-221	EC	5-FU	Decrease	DKK2	[43]
miR-93	BC	Doxorubicin	Decrease	PTEN	[44]
miR-27a	LC	Cisplatin	Decrease	RKIP	[45]
miR-155	BC	Doxorubicin and paclitaxel	Decrease	FOXO3a	[48]
	GC	Paclitaxel	Decrease	GATA3 and TP53INP1	[49]
miR-32-5p	HCC	5-FU	Decrease	PTEN	[50]
miR-509-5p	PC	Gemcitabine	Increase	Vimentin	[53]
miR-138-5p	PC	5-FU	Increase	Vimentin	[54]
miR-30a	PC	Gemcitabine	Increase	Snail	[56]
miR-153	PC	Gemcitabine	Increase	Snail	[57]
miR-363	OC	Cisplatin	Increase	Snail	[58]
miR-34	PC	Gemcitabine	Increase	Slug	[59]
miR-27b	LC	Cisplatin	Increase	Snail	[60]
miR-27b, miR-34a	PC	Docetaxel	Increase	ZEB1	[61]
miR-181a	TSCC	Cisplatin	Increase	Twist	[63]
miR-186	OC	Cisplatin	Increase	Twist	[64]
	GBM	TMZ	Increase	Twist	[65]
miR-708-3p	BC	Doxorubicin	Increase	ZEB1	[66]
miR-218	LC	Cisplatin	Increase	ZEB2, Slug	[67]
miR-200c	LC	Paclitaxel	Increase	Cathepsin L	[69]
miR-200a	BC	Cisplatin	Decrease	TP53INP1, YAP1	[72]
miR-1243	PC	Gemcitabine	Increase	SMAD4	[53]
miR-25-3p	CC	Cisplatin	Increase	Sema4C	[74]
miR-31-3p	CC	Cisplatin	Increase	Sema4C	[75]
miR-296-3p	NPC	Cisplatin	Increase	MK2	[77]
	LC	Cisplatin	Increase	PRKCA	[78]
miR-1294	OC	Cisplatin	Increase	IGF1R	[82]
miR-128-3p	CRC	Oxaliplatin	Increase	Bmi1 and MRP5	[83]
miR-5100	LC	Cisplatin	Decrease	Rab6	[84]
miR-125b	BC	Gemcitabine, Taxol	Decrease	BAK1	[86]
miR-455-3p	EC	Cisplatin	Decrease	NA	[87]
miR-27b	BC	Docetaxel	Increase	ENPP1	[89]
miR-328	CRC	5-FU, HCPT	Increase	ABCG2	[90]
miR-451	CRC	SN38	Increase	ABCB1	[91]
miR-181b	LC	Cisplatin	Increase	Notch2	[93]
miR-365	HCC	Cisplatin	Increase	RAC1	[98]
miR-485	LC	Cisplatin	Increase	CD44	[99]
miR-1246	BC	Docetaxel, epirubicin, gemcitabine	Decrease	Cyclin G2	[100]
miR-9-5p, miR-195-5p, miR-203a-3p	BC	Doxorubicin, Docetaxel	Decrease	ONECUT2	[101]
miR-129-5p	BC	Doxorubicin	Increase	SOX4	[118]
miR-532-3p	CRC	5-FU, cisplatin	Increase	ETS1 and TGM2	[119]
miR-224	CRC	5-FU	Decrease	NA	[124]
miR-145	GBM	TMZ, cisplatin	Increase	Oct4 and Sox2	[127]

*5-FU* 5-fluorocrail, *APC*adenomatous polyposis col, *ABCB1* ABC subfamily B member 1, *ABCG2*ABC subfamily G member 2, *Bak1*BCL2 antagonist/killer 1, *BC*breast cancer, *Bmi1* B lymphoma Mo-MLV insertion region 1 homolog, *CC* Cervical cancer, *CDH1* Cadherin 1, *CLDN1* Claudin 1, *CRC* colorectal cancer, *EC* esophageal cancer, *ENPP1* ectonucleotide pyrophosphatase/phosphodiesterase 1, *ETS1* V-Ets avian erythroblastosis virus E26 oncogene homolog 1, *FOXO3a* Forkhead Box O3, *FZD4* frizzled class receptor 4, *GATA3* GATA binding protein 3, *GBM* Glioblastoma, *GC* gastric cancer, *HCPT* hydroxycamptothecine, *IGF1R* insulin like growth factor 1 receptor, *LC* lung cancer, *MK2* MAPK activated protein kinase 2, *MRP5* multidrug resistant protein 5, *NA* not acquired, *NIN1* Netrin 1, *NPC* Nasopharyngeal carcinoma, *OC* ovarian cancer, *Oct4* Octamer-binding protein 4, *ONECUT2* one cut homeobox 2, *PC* Pancreatic cancer, *PRKCA* Protein kinase C alpha, *RAC1* Ras-related C3 botulinum toxin substrate 1, *Rab6* Ras-related protein Rab-6a, *RKIP* Raf kinase inhibitory protein, *SN38* 7-ethyl-10-hydroxycamptothecin, *SOX* SRY-box transcription factor, *TGM2* transglutaminase 2, *TMZ* temozolomide, *TP53INP1* tumor protein P53 inducible nuclear protein 1, *YAP1* Yes associated protein 1

### MiRNAs facilitate EMT to induce chemotherapy resistance

MiRNAs can contribute to chemoresistance by directly targeting the epithelial markers. For instance, miR-375 induces paclitaxel chemoresistance by directly suppressing E-cadherin in lung cancer [39]. Besides, miR-514b-5p can decrease the expression of E-cadherin to facilitate drug resistance. Intriguingly, despite derived from the identical RNA hairpin, miR-514b-3p plays an opposite role, which reverses the EMT-induced drug resistance [40]. However, how the precursor of miR-514 becomes the mature miR-514b-3p and miR-514b-5p with distinct roles remains to be further investigated. Furthermore, the miR-106b-25 cluster promotes doxorubicin resistance via repressing EP300, a transcriptional activator of E-cadherin [41].

There exist complex signal pathways of the EMT program, which miRNAs participate in. Wnt is a critical signal in regulating EMT-associated chemoresistance. Yu et al. reported miR-125b promoted the EMT process and induced 5-fluorouracil (5-FU) resistance in colorectal cancer through targeting the APC/Wnt/β-catenin pathway. Remarkably, the expression of miR-125b could be upregulated by CXCL12/CXCR4 [42]. In addition, miR-221 enhanced the resistant capacity to 5-FU of esophageal cancer via the Wnt/β-catenin pathway by directly targeting Dickkopf-2 [43]. PTEN, a tumor suppressor, is identified to participate in repressing EMT by inhibiting PI3K/AKT signal. Chu et al. found miR-93 contributed to eliciting EMT and facilitating doxorubicin resistance in breast cancer via the suppression of PTEN [44]. It was observed that miR-27a was dramatically upregulated in cisplatin-resistant lung adenocarcinoma. Mechanism exploration revealed that miR-27a targeted Raf kinase inhibitory protein to rescue Raf signal, which was involved in EMT-induced cisplatin resistance [45].

Exosomes are a subset of extracellular vesicles with a diameter ranging from 40nm to 160nm, which can mediate cell communication in physiological and pathological conditions via transferring specific cargos (nucleic acid or protein) [46]. Recently, it is reported that miRNAs carried by exosomes derived from drug-resistant cells can confer the resistant ability to drug-sensitive ones [47]. For example, CSCs and resistant cancer cells can secrete exosomal miR-155, which induces the EMT process to enhance the resistance to chemotherapy of breast cancer [48]. Besides, exosomal miR-155 was found to promote the EMT and chemoresistant phenotypes in gastric cancer by targeting GATA binding protein 3 and tumor protein p53‑inducible nuclear protein 1 [49]. Fu et al. identified the transmission of multidrug resistance in hepatocellular carcinoma was attributed to exosomal miR-32-5p that inhibited PTEN/PI3K/AKT pathways [50]. On the other hand, exosomes can also regulate drug resistance by changing the transcriptome of cancer cells. Exosomes derived from mesenchymal-like prostate cancer cells confer the recipient cells with the ability to resist enzalutamide, an androgen receptor antagonist. It was observed that the expressions of miR-21, miR-31, and miR-145 were upregulated with the activation of the TGF-β signaling pathway when the recipient cells took up the exosomes [51]. However, the content of exosomes hasn’t been identified. Although EMT-related pathways mediated by miRNAs in drug resistance have been broadly investigated, there remain several key problems about exosomal miRNAs-mediated drug resistance. For example, how the EMT-related miRNAs are loaded into exosomes hasn’t been exhaustively figured out. Furthermore, inhibition of drug resistance-induced exosomal miRNA cannot entirely abolish the resistance, indicating drug resistance is a complex process where other factors may be involved [52].

### MiRNAs inhibit EMT to overcome chemotherapy resistance

There also exist numerous miRNAs that suppress chemoresistance by inhibiting the EMT process. To begin with, miRNA can inhibit mesenchymal markers like vimentin to overcome chemoresistance. Overexpression of miR-509-5p increases the sensitivity to gemcitabine in pancreatic cancer by targeting vimentin [53]. Furthermore, miR-138-5p that can be downregulated by TGF-β also targets vimentin to enhance the chemosensitivity to 5-FU in pancreatic cancer [54, 55].

Additionally, miRNAs can inhibit the EMT-TFs to overcome drug resistance. It was reported that miR-30a, miR-153, and miR-363 all targeted Snail to enhance chemotherapy sensitivity [56–58]. In addition, miR-34 and miR-27b were found to increase chemotherapy sensitivity through targeting Slug and Snail, respectively [59, 60]. Meanwhile, Zhang et al. identified miR-34a and miR-27b could enhance docetaxel sensitivity via inhibiting ZEB1 in prostate cancer [61]. Notably, hypoxia can repress the expression of miR-34a, attenuating its antitumor effect [62]. Thus, improving the hypoxic microenvironment will be a novel strategy to overcome the therapy resistance. Twist, another key EMT-TF, can be suppressed by miR-181a with the increased cisplatin sensitivity simultaneously [63]. Moreover, miR-186 was reported as a chemotherapy sensitizer through targeting Twist in not only ovarian cancer but also glioblastoma [64, 65]. As for ZEB, miR-708-3p acts as a ZEB1 suppressor to increase drug resistance in breast cancer [66]. Similarly, Shi et al. identified miR-218 served as a drug sensitizer through directly targeting ZEB2 in lung cancer, which provided a potential therapeutic strategy [67]. However, the therapeutic effect should be further evaluated because EMT-TFs often act cooperatively and are modulated by other upstream regulative EMT-TFs.

Through targeting the EMT-related signal pathways, miRNAs can exert their anti-chemoresistant functions. Generally, the miR-200 family, containing miR-141, miR-200a, miR-200b, miR-200c, and miR-429, serves as a tumor suppressor in manifold cancer types. It is well known that the miR-200 family decreases the expression of TGF-β to repress the EMT process and drug resistance [68]. In addition to the TGF-β/SMAD pathways, miR-200c can overcome chemoresistance by reducing Cathepsin L that has been regarded as a potential target in cancer treatment [69, 70]. Moreover, the miR-200c/c-myc negative regulatory feedback loop is crucial for the EMT process and CSC properties as well as drug sensitivity [71]. Nevertheless, there exists an opposite voice about the role of miR-200. For instance, Yu et al. found miR-200a confer the sensitive breast cancer cells with the chemoresistant ability through antagonizing tumor protein p53‑inducible nuclear protein 1 and YAP1 [72]. TGF-β signal is also regulated by other miRNAs. Recently, miR-1243 was found to increase the expression of E-cadherin and reverse drug resistance via suppressing SMAD4 [53]. Semaphorin 4 C plays a key role in promoting TGF-β-induced EMT [73]. Overexpression of miR-25-3p and miR-31-3p can target Semaphorin 4 C to reverse EMT in cisplatin-resistance cervical cancer cells [74, 75]. TGF-β can affect the expression of miRNAs in turn, to regulate the EMT, resulting in the change of drug sensitivity. For example, TGF-β can suppress miR-499a to induce drug resistance, which inhibits EMT in osteosarcoma through targeting SH3K binding protein 1 [76]. Furthermore, the Ras/Raf signal pathway has a significant influence on EMT and chemoresistance, too. It was found that miR-296-3p could suppress the expression of MAPK activated protein kinase 2 to inhibit the Ras/Braf/Erk/Mek/c-Myc pathway, ultimately reversing the chemoresistance in NPC [77]. In lung adenocarcinoma, miR-296-3p also contributes to the inhibition of Ras, leading to the increased chemotherapy sensitivity [78]. It is also reported that miR-296-5p inhibited stemness potency and EMT via BRM/SWI2-related gene 1 and neuregulin 1, respectively [79]. Intriguingly, miR-95 knockdown could repress EMT and CSCs phenotype through dual specificity phosphatase 5-dependent MAPK pathway [80]. Insulin-like growth factor 1 receptor (IGF1R) can activate PI3K/ATK and Ras/Raf signal pathways to participate in the generation of drug resistance, respectively [81]. Zhang et al. showed miR-1294 could bind the 3’UTR of IGF1R to prevent the EMT program and reverse cisplatin resistance in ovarian cancer [82].

Interestingly, exosomal miRNAs derived from normal cells are able to reverse drug resistance. For example, derived from intestinal epithelial cells, exosomal miR-128-3p shows the outstanding capability to increase chemosensitivity to oxaliplatin in colorectal cancer. Mechanically, miR-128-3p can prevent the efflux of oxaliplatin via repressing the drug transporter multidrug resistant protein 5 and reverse the oxaliplatin-induced EMT [83].

### MiRNAs promote CSCs features to induce chemotherapy resistance

In addition to regulating the EMT-mediated drug resistance, miRNAs can directly affect the CSCs status to modulate drug sensitivity. As mentioned above, CSCs-mediated chemoresistance is attributed to anti-apoptosis, activate DNA repair, and increased drug efflux. Rab6, a small GTP-binding protein, belongs to the Ras superfamily, which is regarded as a pro-apoptotic factor. Overexpression of miR-5100 was observed in lung CSCs, which can inhibit cisplatin-induced mitochondrial apoptosis by directly targeting Rab6 [84]. Apart from promoting EMT that is aforementioned, miR-125b can directly regulate CSCs phenotype to induce chemoresistance. Wang et al. observed the phenomenon that miR-125b confers the chemoresistant capability of breast cancer by maintaining CSCs state [85]. The further study identified BCL2 antagonist/killer 1 as the direct target of miR-125b in such a phenomenon [86].

Owing to the interactive signal pathways between EMT and CSCs, miRNAs regulating EMT-associated signals also have a significant influence on the generation of CSCs characteristics. Liu et al. found miR-455-3p as an oncomiR can maintain CSCs state to reinforce the chemoresistance in esophageal cancer by activating Wnt/β-catenin and TGF-β signaling. Remarkably, treatment with the miR-455-3p antagomir significantly sensitized esophageal cancer *in vitro*, which provided a novel therapeutic strategy for esophageal cancer [87]. However, the therapeutic effect *in vitro* needs to be evaluated and more efforts should be put into the translation to clinical application.

### MiRNAs inhibit CSCs to overcome chemotherapy resistance

In CSCs, one of the most important mechanisms of drug resistance is the overexpression of the ATP-binding cassette (ABC) family, which transports drugs out of cells, protecting the cells from cytotoxicity [88]. MiRNAs can modulate the expression of the ABC family to affect the resistant phenotype in CSCs. For instance, miR-27b indirectly represses ABCG2 by affecting its localization on the cell surface. As a result, breast cancer patients with the downregulation of miR-27b was inclined to relapse due to the emergence of a small group of cells harboring CSCs properties [89]. In addition, miR-328 and miR-451 reverse the chemotherapy resistance by directly targeting ABCG2 and ABC subfamily B member 1 in CSCs, respectively [90, 91].

MiRNAs can regulate the stemness-associated signal pathways to overcome chemoresistance, in which the Notch signal plays a pivotal role [92]. Notch signal can contribute to the reduced sensitivity to cisplatin and the suppression of CSCs features while it is repressed by miR-181b in lung cancer [93]. Similarly, miR-136 enhances the antitumor effect of paclitaxel in ovarian cancer by decreasing Notch3 [94]. Another crucial pathway concerning the generation of CSCs is the Ras signaling pathway. Upregulation of miR-17-92 cluster can facilitate the exhaustion of pancreatic CSCs by reducing Ras and cyclin dependent kinase inhibitor 1 C, resulting in the reverse of chemoresistance [95]. Ras-related C3 botulinum toxin substrate 1 (RAC1) is also a subfamily of the Ras superfamily, which mediates intercellular adhesion, cell cycle, and epithelial differentiation [96]. Upregulation of miR-194 and miR-365 targeting RAC1 inhibits liver CSCs expansion, leading to the increased sensitivity to sorafenib and cisplatin [97, 98]. In addition to regulating the complicated signals, miRNAs are able to diminish the number of CSCs more directly—targeting the hallmarks of CSCs. For example, stemness features and CSC population were repressed by miR-485/CD44 axis in cisplatin-resistant lung cancer cells [99].

Exosome-loaded miRNAs are also vital to spread drug resistance to those sensitive cancer cells. Exosomal miR-1246 is related to stem-like traits and chemoresistance, which could serve as a prognostic predictor in breast cancer patients. Mechanically, miR-1246 exert its oncogenic role by inhibiting cyclin-G2 [100]. MiR-9-5p, miR-195-5p, and miR-203a-3p carried by exosomes all target One Cut Homeobox 2 (ONECUT2) to enhance the stemness of breast cancer. Notably, the upregulations of these exosomal miRNAs are induced by chemotherapy [101]. Additionally, gemcitabine-resistant pancreatic CSCs disseminate the resistant phenotype by delivering exosomal miR-210 [102]. Importantly, the upregulation of miR-210 is elicited by hypoxia [103], which indicates inhibiting exosomal miR-210 and improving the hypoxic microenvironment simultaneously may achieve a better therapeutic effect.

## The therapeutic strategy of inhibiting EMT and CSCs to overcome chemoresistance

Since the roles of EMT and CSCs in chemoresistance are gradually determined, a promising therapeutic strategy for overcoming chemoresistance is to repress EMT and CSCs. The rapid progress of CSC-associated drug resistance is attributed to the advanced technique of identifying and isolating CSCs, which makes researchers analyze the distinct drug sensitivities between CSCs and non-CSCs. Nevertheless, the idea of precisely targeting CSCs is faced with several challenges. On one hand, the reliable hallmarks to accurately identify CSCs in bulk cancers are still insufficient. On the other hand, how to ensure the stem cells from normal tissues escape the cytotoxicity of chemotherapeutics remains to be solved. Fortunately, benefiting from the interplay between CSCs and the EMT program, a substituted therapy of targeting EMT seem feasible due to the existence of definite biomarkers and signal pathways.

Given that the EMT is dynamic and requires a certain process, the therapeutic approaches can be primarily divided into preventing EMT initiation, eliminating the cells undergoing EMT, and activating the opposite process of EMT—mesenchymal-epithelial transition (MET) [23].

TGF-β signal is among the best-characterized pathways in inducing EMT. Therefore, the blockage of the TGF-β pathway may be an effective approach in preventing the initiation of EMT and overcome drug resistance. As expected, several TGF-β inhibitors are undergoing clinical trials and achieve a certain therapeutic effect [104, 105]. However, TGF-β has a broad function in physiological and pathological conditions, which is not merely limited to affecting EMT. Hence, whether TGF-β inhibitors influence other biological processes need to be further evaluated. In addition, TGF-β serves as a tumor suppressor in early-stage cancer and it is cautious to choose the optimal medication time [106].

Another approach is to improve the tumor microenvironment, which also contributes to the activation of EMT. The conventional method is to inhibit tumor-associated inflammation and hypoxia. In recent years, with the gradual insight into the role of cancer-associated fibroblast and tumor-associated macrophage in EMT-associated metastasis, the strategy of targeting these cells has drawn considerable attention, especially targeting the exosomes derived from them [107, 108]. However, EMT triggered by these components of the tumor microenvironment remains under investigation.

As for eliminating the cells undergoing EMT, the initial attempt is to repress the EMT biomarkers. Kaschula et al. found ajoene derived from garlic could disrupt the vimentin filament network to exert the anti-metastatic function, while the role of overcoming drug resistance needs to be conducted [109]. However, the mesenchymal markers are also widely expressed in normal mesenchymal cells, leading to the potential off-tumor toxicities. Recently, Lou et al. reported the c-Src inhibitor could selectively target the overexpressed vimentin in triple-negative breast cancer, which may provide a new solution to this problem [110]. Another approach is to repress the specifically expressed gene in the EMT program. It was found that Axl was significantly upregulated during the process of EMT and knockdown of Axl by siRNA inhibited the metastasis and increased the overall survival in breast cancer [111]. In 2013, the first Axl inhibitor BGB324 entered clinical trials [112]. Recently, the recruitment of a phase II, multicenter clinical trial of BGB324 combined with pembrolizumab in treating triple negative breast cancer has been completed (NCT03184558).

From the perspective of the principle of EMT, reversing EMT to MET seems to be effective in overcoming drug resistance. It was reported the increased expression of intracellular second messenger cAMP induced MET via activating protein kinase A [113]. This study revealed a role of protein kinase A in maintaining and reinforcing the epithelial state, which suggested protein kinase A may act as a new therapeutic target. However, cancer metastasis is likely associated with the re-epithelization of mesenchymal cells or CSCs, which is aforementioned. Thus, choosing the proper time of this strategy needs to be particularly careful otherwise it may be a pro-metastatic factor.

Since the EMT is regulated by miRNAs, the exogenous introduction of miRNA mimics or antagomir may enhance the drug sensitivity. The downregulation of miR-129-5p and miR-532-3p are associated with the poor prognosis in manifold cancer types, which are involved in the EMT program [114–117]. Hence, Luan et al. and Gu et al. used miR-129-5p and miR-532-3p mimics, respectively, to enhance the chemosensitivity *in vivo* [118, 119]. Recently, miR-147, miR-335, miR-1976, and miR-4319 were identified as tumor suppressor miRNAs for inhibiting EMT and CSCs simultaneously [120–123]. However, their roles in reversing drug resistance have not been demonstrated clearly, which need to be further explored. On the contrary, miR-224 is responsible for the poor response of 5-FU, and silencing miR-224 by antagomir achieves the desired effect in colorectal cancer cells [124]. Nevertheless, miRNAs have broad functions due to their tissue specificity and target gene diversity. It remains unknown whether the inhibition of a specific miRNA will have an influence on other signal pathways. Moreover, the efficacy of RNA interference is likely to be reduced *in vitro* study due to the degradation and off-target effects [125, 126]. Thus, it is important to select a proper delivery vehicle. Yang et al. used polyurethane-short branch polyethyleneimine as the vehicle to deliver miR-145 that can inhibit stem-like features and chemoresistance simultaneously [127]. However, the chemosynthetic carrier is faced with the challenge of biocompatibility *in vitro.*

Recently, delivering functional small RNAs by exosomes is a decent approach to solve these problems. Exosomes are stable and of biological origin, which can protect the cargos from being degraded [128]. Furthermore, the ligand/receptor on the exosome membrane is usually modified for better targetability. For instance, IL-3R is overexpressed on chronic myelogenous leukemia blasts [129], so Bellavia et al. coated a fragment of IL-3 on exosomes to precisely target CML cells. It has been shown the engineered exosomes carrying BCR-ABL siRNA can significantly inhibit cell growth [130]. However, the exosome-based delivering strategy is also faced with quite a few defects. It is urgent to develop techniques to realize the large-scale preparation of therapeutic exosomes. Besides, the therapeutic effect should be further verified in a large number of clinical studies.

## Conclusion

In summary, the deep understanding of the link between the EMT program and the CSCs status provides us with new insight into therapy resistance. Cancer cells undergoing EMT acquire the CSCs properties, which are regulated by multiple factors, such as EMT-TFs and various signal pathways. In this process, miRNAs play a pivotal role. Importantly, targeting EMT and CSCs will be a promising therapeutic strategy in overcoming chemoresistance. Furthermore, inhibiting the EMT-induced miRNAs or introducing the EMT-suppressed miRNAs is also attractive. However, applying these therapeutic approaches to clinical practice remains a long way to go. More efforts should be put into identifying cancer type specific miRNAs and refining deleivery approaches for miRNAs into cancer cells.

## Data Availability

Data sharing not applicable to this article as no datasets were generated or analyzed during the current study.

## Abbreviations

5-FU: 5-fluorouracil
ABC: ATP-binding cassette
ABCG2: ATP-binding cassette subfamily G member 2
ALDH: Aldehyde dehydrogenase
CSCs: Cancer stem cells
EMT: Epithelial-to-mesenchymal transition
EMT-TFs: EMT-inducing transcription factors
IGF1R: Insulin-like growth factor 1 receptor
MET: Mesenchymal-epithelial transition
MiRNAs: MicroRNAs
RAC1: Ras-related C3 botulinum toxin substrate 1
YAP1: Yes-associated protein 1

## References

[1] Rahimi M, Sharifi-Zarchi A, Firouzi J, Azimi M, Zarghami N, Alizadeh E (2019). An integrated analysis to predict micro-RNAs targeting both stemness and metastasis in breast cancer stem cells. J Cell Mol Med.

[2] Wu J, Cang S, Liu C, Ochiai W, Chiao JW (2020). Development of human prostate cancer stem cells involves epigenomic alteration and PI3K/AKT pathway activation. Exp Hematol Oncol.

[3] Wu HJ, Chu PY (2019). Role of Cancer Stem Cells in Cholangiocarcinoma and Therapeutic Implications. Int J Mol Sci.

[4] Xu H, Niu M, Yuan X, Wu K, Liu A (2020). CD44 as a tumor biomarker and therapeutic target. Exp Hematol Oncol.

[5] Liu YC, Yeh CT, Lin KH (2020). Cancer Stem Cell Functions in Hepatocellular Carcinoma and Comprehensive Therapeutic Strategies. Cells.

[6] Diepenbruck M, Christofori G (2016). Epithelial-mesenchymal transition (EMT) and metastasis: yes, no, maybe?. Curr Opin Cell Biol.

[7] Ling Z, Cheng B, Tao X. Epithelial-to-mesenchymal transition in oral squamous cell carcinoma: Challenges and opportunities. Int J Cancer. 2020; Oct 22. Online ahead of print. 10.1002/ijc.33352 33091960

[8] Guo H, Ingolia NT, Weissman JS, Bartel DP (2010). Mammalian microRNAs predominantly act to decrease target mRNA levels. Nature.

[9] Svoronos AA, Engelman DM, Slack FJ (2016). OncomiR or Tumor Suppressor? The Duplicity of MicroRNAs in Cancer. Cancer Res.

[10] Liu Y, Cheng Z, Pang Y, Cui L, Qian T, Quan L (2019). Role of microRNAs, circRNAs and long noncoding RNAs in acute myeloid leukemia. J Hematol Oncol.

[11] Yi M, Xu L, Jiao Y, Luo S, Li A, Wu K (2020). The role of cancer-derived microRNAs in cancer immune escape. J Hematol Oncol.

[12] Wang W, Han Y, Jo HA, Lee J, Song YS (2020). Non-coding RNAs shuttled via exosomes reshape the hypoxic tumor microenvironment. J Hematol Oncol.

[13] Dong P, Konno Y, Watari H, Hosaka M, Noguchi M, Sakuragi N (2014). The impact of microRNA-mediated PI3K/AKT signaling on epithelial-mesenchymal transition and cancer stemness in endometrial cancer. J Transl Med.

[14] Mittal V (2018). Epithelial Mesenchymal Transition in Tumor Metastasis. Annu Rev Pathol.

[15] Mareel M, Vleminckx K, Vermeulen S, Bracke M, Van Roy F (1992). E-cadherin expression: a counterbalance for cancer cell invasion. Bull Cancer.

[16] Xu J, Lamouille S, Derynck R (2009). TGF-beta-induced epithelial to mesenchymal transition. Cell Res.

[17] Lamouille S, Xu J, Derynck R (2014). Molecular mechanisms of epithelial-mesenchymal transition. Nat Rev Mol Cell Biol.

[18] De Craene B, Berx G (2013). Regulatory networks defining EMT during cancer initiation and progression. Nat Rev Cancer.

[19] Taube JH, Herschkowitz JI, Komurov K, Zhou AY, Gupta S, Yang J (2010). Core epithelial-to-mesenchymal transition interactome gene-expression signature is associated with claudin-low and metaplastic breast cancer subtypes. Proc Natl Acad Sci U S A.

[20] Hill L, Browne G, Tulchinsky E (2013). ZEB/miR-200 feedback loop: at the crossroads of signal transduction in cancer. Int J Cancer.

[21] Nie D, Fu J, Chen H, Cheng J, Fu J (2019). Roles of MicroRNA-34a in Epithelial to Mesenchymal Transition, Competing Endogenous RNA Sponging and Its Therapeutic Potential. Int J Mol Sci.

[22] García-Heredia JM, Carnero A (2020). Role of Mitochondria in Cancer Stem Cell Resistance. Cells.

[23] Shibue T, Weinberg RA (2017). EMT, CSCs, and drug resistance: the mechanistic link and clinical implications. Nat Rev Clin Oncol.

[24] Pan Q, Li Q, Liu S, Ning N, Zhang X, Xu Y (2015). Concise Review: Targeting Cancer Stem Cells Using Immunologic Approaches. Stem Cells.

[25] López de Andrés J, Griñán-Lisón C, Jiménez G, Marchal JA (2020). Cancer stem cell secretome in the tumor microenvironment: a key point for an effective personalized cancer treatment. J Hematol Oncol.

[26] Mani SA, Guo W, Liao MJ, Eaton EN, Ayyanan A, Zhou AY (2008). The epithelial-mesenchymal transition generates cells with properties of stem cells. Cell.

[27] Lathia JD, Liu H (2017). Overview of Cancer Stem Cells and Stemness for Community Oncologists. Target Oncol.

[28] Zhu P, Wang Y, Wu J, Huang G, Liu B, Ye B (2016). LncBRM initiates YAP1 signalling activation to drive self-renewal of liver cancer stem cells. Nat Commun.

[29] French R, Pauklin S. Epigenetic regulation of cancer stem cell formation and maintenance. Int J Cancer. 2020. 10.1002/ijc.33398 PMC8246550 33197277

[30] Sun HR, Wang S, Yan SC, Zhang Y, Nelson PJ, Jia HL (2019). Therapeutic Strategies Targeting Cancer Stem Cells and Their Microenvironment. Front Oncol.

[31] Kyriazi AA, Papiris E, Kitsos Kalyvianakis K, Sakellaris G, Baritaki S. Dual Effects of Non-Coding RNAs (ncRNAs) in Cancer Stem Cell Biology. Int J Mol Sci. 2020;21(18). 10.3390/ijms21186658 PMC7556003 32932969

[32] Su HT, Weng CC, Hsiao PJ, Chen LH, Kuo TL, Chen YW (2013). Stem cell marker nestin is critical for TGF-β1-mediated tumor progression in pancreatic cancer. Mol Cancer Res.

[33] Dongre A, Weinberg RA (2019). New insights into the mechanisms of epithelial-mesenchymal transition and implications for cancer. Nat Rev Mol Cell Biol.

[34] Huber MA, Kraut N, Beug H (2005). Molecular requirements for epithelial-mesenchymal transition during tumor progression. Curr Opin Cell Biol.

[35] Scheel C, Eaton EN, Li SH, Chaffer CL, Reinhardt F, Kah KJ (2011). Paracrine and autocrine signals induce and maintain mesenchymal and stem cell states in the breast. Cell.

[36] Ni T, Li XY, Lu N, An T, Liu ZP, Fu R (2016). Snail1-dependent p53 repression regulates expansion and activity of tumour-initiating cells in breast cancer. Nat Cell Biol.

[37] Garg M (2015). Targeting microRNAs in epithelial-to-mesenchymal transition-induced cancer stem cells: therapeutic approaches in cancer. Expert Opin Ther Targets.

[38] Wang WT, Han C, Sun YM, Chen TQ, Chen YQ (2019). Noncoding RNAs in cancer therapy resistance and targeted drug development. J Hematol Oncol.

[39] Shen Y, Zhou J, Li Y, Ye F, Wan X, Lu W (2014). miR-375 mediated acquired chemo-resistance in cervical cancer by facilitating EMT. PLoS One.

[40] Ren LL, Yan TT, Shen CQ, Tang JY, Kong X, Wang YC (2018). The distinct role of strand-specific miR-514b-3p and miR-514b-5p in colorectal cancer metastasis. Cell Death Dis.

[41] Zhou Y, Hu Y, Yang M, Jat P, Li K, Lombardo Y (2014). The miR-106b ~ 25 cluster promotes bypass of doxorubicin-induced senescence and increase in motility and invasion by targeting the E-cadherin transcriptional activator EP300. Cell Death Differ.

[42] Yu X, Shi W, Zhang Y, Wang X, Sun S, Song Z (2017). CXCL12/CXCR4 axis induced miR-125b promotes invasion and confers 5-fluorouracil resistance through enhancing autophagy in colorectal cancer. Sci Rep.

[43] Wang Y, Zhao Y, Herbst A, Kalinski T, Qin J, Wang X (2016). miR-221 Mediates Chemoresistance of Esophageal Adenocarcinoma by Direct Targeting of DKK2 Expression. Ann Surg.

[44] Chu S, Liu G, Xia P, Chen G, Shi F, Yi T (2017). miR-93 and PTEN: Key regulators of doxorubicin-resistance and EMT in breast cancer. Oncol Rep.

[45] Li J, Wang Y, Song Y, Fu Z, Yu W (2014). miR-27a regulates cisplatin resistance and metastasis by targeting RKIP in human lung adenocarcinoma cells. Mol Cancer.

[46] Li S, Yi M, Dong B, Tan X, Luo S, Wu K. The role of exosomes in liquid biopsy for cancer diagnosis and prognosis prediction. Int J Cancer. 2020; Nov 12. Online ahead of print. 10.1002/ijc.33386 PMC8049049 33180334

[47] Li S, Yi M, Dong B, Jiao Y, Luo S, Wu K (2020). The roles of exosomes in cancer drug resistance and its therapeutic application. Clin Transl Med.

[48] Santos JC, Lima NDS, Sarian LO, Matheu A, Ribeiro ML, Derchain SFM (2018). Exosome-mediated breast cancer chemoresistance via miR-155 transfer. Sci Rep.

[49] Wang M, Qiu R, Yu S, Xu X, Li G, Gu R (2019). Paclitaxel–resistant gastric cancer MGC–803 cells promote epithelial–to–mesenchymal transition and chemoresistance in paclitaxel–sensitive cells via exosomal delivery of miR–155–5p. Int J Oncol.

[50] Fu X, Liu M, Qu S, Ma J, Zhang Y, Shi T (2018). Exosomal microRNA-32-5p induces multidrug resistance in hepatocellular carcinoma via the PI3K/Akt pathway. J Exp Clin Cancer Res.

[51] El-Sayed IY, Daher A, Destouches D, Firlej V, Kostallari E, Maillé P (2017). Extracellular vesicles released by mesenchymal-like prostate carcinoma cells modulate EMT state of recipient epithelial-like carcinoma cells through regulation of AR signaling. Cancer Lett.

[52] Zhu X, Shen H, Yin X, Yang M, Wei H, Chen Q (2019). Macrophages derived exosomes deliver miR-223 to epithelial ovarian cancer cells to elicit a chemoresistant phenotype. J Exp Clin Cancer Res.

[53] Hiramoto H, Muramatsu T, Ichikawa D, Tanimoto K, Yasukawa S, Otsuji E (2017). miR-509-5p and miR-1243 increase the sensitivity to gemcitabine by inhibiting epithelial-mesenchymal transition in pancreatic cancer. Sci Rep.

[54] Yu C, Wang M, Chen M, Huang Y, Jiang J (2015). Upregulation of microRNA–138–5p inhibits pancreatic cancer cell migration and increases chemotherapy sensitivity. Mol Med Rep.

[55] Zhang F, Li T, Han L, Qin P, Wu Z, Xu B (2018). TGFβ1-induced down-regulation of microRNA-138 contributes to epithelial-mesenchymal transition in primary lung cancer cells. Biochem Biophys Res Commun.

[56] Wang T, Chen G, Ma X, Yang Y, Chen Y, Peng Y (2019). MiR-30a regulates cancer cell response to chemotherapy through SNAI1/IRS1/AKT pathway. Cell Death Dis.

[57] Liu F, Liu B, Qian J, Wu G, Li J, Ma Z (2017). miR-153 enhances the therapeutic effect of gemcitabine by targeting Snail in pancreatic cancer. Acta Biochim Biophys Sin (Shanghai).

[58] Cao L, Wan Q, Li F, Tang CE (2018). MiR-363 inhibits cisplatin chemoresistance of epithelial ovarian cancer by regulating snail-induced epithelial-mesenchymal transition. BMB Rep.

[59] Zhang QA, Yang XH, Chen D, Yan X, Jing FC, Liu HQ (2018). miR-34 increases in vitro PANC-1 cell sensitivity to gemcitabine via targeting Slug/PUMA. Cancer Biomark.

[60] Zhang J, Hua X, Qi N, Han G, Yu J, Yu Y (2020). MiR-27b suppresses epithelial-mesenchymal transition and chemoresistance in lung cancer by targeting Snail1. Life Sci.

[61] Zhang G, Tian X, Li Y, Wang Z, Li X, Zhu C (2018). miR-27b and miR-34a enhance docetaxel sensitivity of prostate cancer cells through inhibiting epithelial-to-mesenchymal transition by targeting ZEB1. Biomed Pharmacother.

[62] Li H, Rokavec M, Jiang L, Horst D, Hermeking H (2017). Antagonistic Effects of p53 and HIF1A on microRNA-34a Regulation of PPP1R11 and STAT3 and Hypoxia-induced Epithelial to Mesenchymal Transition in Colorectal Cancer Cells. Gastroenterology.

[63] Liu M, Wang J, Huang H, Hou J, Zhang B, Wang A (2013). miR-181a-Twist1 pathway in the chemoresistance of tongue squamous cell carcinoma. Biochem Biophys Res Commun.

[64] Zhu X, Shen H, Yin X, Long L, Xie C, Liu Y (2016). miR-186 regulation of Twist1 and ovarian cancer sensitivity to cisplatin. Oncogene.

[65] Xiong Y, Chen R, Wang L, Wang S, Tu Y, Zhu L (2019). Downregulation of miR–186 promotes the proliferation and drug resistance of glioblastoma cells by targeting Twist1. Mol Med Rep.

[66] Lee JW, Guan W, Han S, Hong DK, Kim LS, Kim H (2018). MicroRNA-708-3p mediates metastasis and chemoresistance through inhibition of epithelial-to-mesenchymal transition in breast cancer. Cancer Sci.

[67] Shi ZM, Wang L, Shen H, Jiang CF, Ge X, Li DM (2017). Downregulation of miR-218 contributes to epithelial-mesenchymal transition and tumor metastasis in lung cancer by targeting Slug/ZEB2 signaling. Oncogene.

[68] Wang Y, Wu Z, Hu L (2018). The regulatory effects of metformin on the [SNAIL/miR-34]:[ZEB/miR-200] system in the epithelial-mesenchymal transition(EMT) for colorectal cancer(CRC). Eur J Pharmacol.

[69] Zhao YF, Han ML, Xiong YJ, Wang L, Fei Y, Shen X (2018). A miRNA-200c/cathepsin L feedback loop determines paclitaxel resistance in human lung cancer A549 cells in vitro through regulating epithelial-mesenchymal transition. Acta Pharmacol Sin.

[70] Lankelma JM, Voorend DM, Barwari T, Koetsveld J, Van der Spek AH, De Porto AP (2010). Cathepsin L, target in cancer treatment?. Life Sci.

[71] Yang J, Wu SP, Wang WJ, Jin ZR, Miao XB, Wu Y (2020). A novel miR-200c/c-myc negative regulatory feedback loop is essential to the EMT process, CSC biology and drug sensitivity in nasopharyngeal cancer. Exp Cell Res.

[72] Yu SJ, Yang L, Hong Q, Kuang XY, Di GH, Shao ZM (2018). MicroRNA-200a confers chemoresistance by antagonizing TP53INP1 and YAP1 in human breast cancer. BMC Cancer.

[73] Zeng R, Han M, Luo Y, Li C, Pei G, Liao W (2011). Role of Sema4C in TGF-β1-induced mitogen-activated protein kinase activation and epithelial-mesenchymal transition in renal tubular epithelial cells. Nephrol Dial Transplant.

[74] Song J, Li Y (2017). miR-25-3p reverses epithelial-mesenchymal transition via targeting Sema4C in cisplatin-resistance cervical cancer cells. Cancer Sci.

[75] Jing L, Bo W, Yourong F, Tian W, Shixuan W, Mingfu W (2019). Sema4C mediates EMT inducing chemotherapeutic resistance of miR-31-3p in cervical cancer cells. Sci Rep.

[76] Wang T, Wang D, Zhang L, Yang P, Wang J, Liu Q (2019). The TGFβ-miR-499a-SHKBP1 pathway induces resistance to EGFR inhibitors in osteosarcoma cancer stem cell-like cells. J Exp Clin Cancer Res.

[77] Deng X, Liu Z, Liu X, Fu Q, Deng T, Lu J (2018). miR-296-3p Negatively Regulated by Nicotine Stimulates Cytoplasmic Translocation of c-Myc via MK2 to Suppress Chemotherapy Resistance. Mol Ther.

[78] Fu Q, Song X, Liu Z, Deng X, Luo R, Ge C (2017). miRomics and Proteomics Reveal a miR-296-3p/PRKCA/FAK/Ras/c-Myc Feedback Loop Modulated by HDGF/DDX5/β-catenin Complex in Lung Adenocarcinoma. Clin Cancer Res.

[79] Shi DM, Shi XL, Xing KL, Zhou HX, Lu LL, Wu WZ (2020). miR-296-5p suppresses stem cell potency of hepatocellular carcinoma cells via regulating Brg1/Sall4 axis. Cell Signal.

[80] Du M, Zhuang Y, Tan P, Yu Z, Zhang X, Wang A (2020). microRNA-95 knockdown inhibits epithelial-mesenchymal transition and cancer stem cell phenotype in gastric cancer cells through MAPK pathway by upregulating DUSP5. J Cell Physiol.

[81] Hakuno F, Takahashi SI (2018). IGF1 receptor signaling pathways. J Mol Endocrinol.

[82] Zhang Y, Huang S, Guo Y, Li L (2018). MiR-1294 confers cisplatin resistance in ovarian Cancer cells by targeting IGF1R. Biomed Pharmacother.

[83] Liu T, Zhang X, Du L, Wang Y, Liu X, Tian H (2019). Exosome-transmitted miR-128-3p increase chemosensitivity of oxaliplatin-resistant colorectal cancer. Mol Cancer.

[84] Yang L, Lin Z, Wang Y, Gao S, Li Q, Li C (2018). MiR-5100 increases the cisplatin resistance of the lung cancer stem cells by inhibiting the Rab6. Mol Carcinog.

[85] Wang HJ, Guo YQ, Tan G, Dong L, Cheng L, Li KJ (2013). miR-125b regulates side population in breast cancer and confers a chemoresistant phenotype. J Cell Biochem.

[86] Liu Z, Liu H, Desai S, Schmitt DC, Zhou M, Khong HT (2013). miR-125b functions as a key mediator for snail-induced stem cell propagation and chemoresistance. J Biol Chem.

[87] Liu A, Zhu J, Wu G, Cao L, Tan Z, Zhang S (2017). Antagonizing miR-455-3p inhibits chemoresistance and aggressiveness in esophageal squamous cell carcinoma. Mol Cancer.

[88] Sarkadi B, Homolya L, Szakács G, Váradi A (2006). Human multidrug resistance ABCB and ABCG transporters: participation in a chemoimmunity defense system. Physiol Rev.

[89] Takahashi RU, Miyazaki H, Takeshita F, Yamamoto Y, Minoura K, Ono M (2015). Loss of microRNA-27b contributes to breast cancer stem cell generation by activating ENPP1. Nat Commun.

[90] Xu XT, Xu Q, Tong JL, Zhu MM, Nie F, Chen X (2012). MicroRNA expression profiling identifies miR-328 regulates cancer stem cell-like SP cells in colorectal cancer. Br J Cancer.

[91] Bitarte N, Bandres E, Boni V, Zarate R, Rodriguez J, Gonzalez-Huarriz M (2011). MicroRNA-451 is involved in the self-renewal, tumorigenicity, and chemoresistance of colorectal cancer stem cells. Stem Cells.

[92] Selim JH, Shaheen S, Sheu WC, Hsueh CT (2019). Targeted and novel therapy in advanced gastric cancer. Exp Hematol Oncol.

[93] Wang X, Meng Q, Qiao W, Ma R, Ju W, Hu J (2018). miR-181b/Notch2 overcome chemoresistance by regulating cancer stem cell-like properties in NSCLC. Stem Cell Res Ther.

[94] Jeong JY, Kang H, Kim TH, Kim G, Heo JH, Kwon AY (2017). MicroRNA-136 inhibits cancer stem cell activity and enhances the anti-tumor effect of paclitaxel against chemoresistant ovarian cancer cells by targeting Notch3. Cancer Lett.

[95] Cioffi M, Trabulo SM, Sanchez-Ripoll Y, Miranda-Lorenzo I, Lonardo E, Dorado J (2015). The miR-17-92 cluster counteracts quiescence and chemoresistance in a distinct subpopulation of pancreatic cancer stem cells. Gut.

[96] Abdrabou A, Wang Z (2018). Post-Translational Modification and Subcellular Distribution of Rac1: An Update. Cells.

[97] Ran RZ, Chen J, Cui LJ, Lin XL, Fan MM, Cong ZZ (2019). miR-194 inhibits liver cancer stem cell expansion by regulating RAC1 pathway. Exp Cell Res.

[98] Jiang ZB, Ma BQ, Liu SG, Li J, Yang GM, Hou YB (2019). miR-365 regulates liver cancer stem cells via RAC1 pathway. Mol Carcinog.

[99] Jiang P, Xu C, Chen L, Chen A, Wu X, Zhou M (2018). EGCG inhibits CSC-like properties through targeting miR-485/CD44 axis in A549-cisplatin resistant cells. Mol Carcinog.

[100] Li XJ, Ren ZJ, Tang JH, Yu Q (2017). Exosomal MicroRNA MiR-1246 Promotes Cell Proliferation, Invasion and Drug Resistance by Targeting CCNG2 in Breast Cancer. Cell Physiol Biochem.

[101] Shen M, Dong C, Ruan X, Yan W, Cao M, Pizzo D (2019). Chemotherapy-Induced Extracellular Vesicle miRNAs Promote Breast Cancer Stemness by Targeting ONECUT2. Cancer Res.

[102] Yang Z, Zhao N, Cui J, Wu H, Xiong J, Peng T (2020). Exosomes derived from cancer stem cells of gemcitabine-resistant pancreatic cancer cells enhance drug resistance by delivering miR-210. Cell Oncol (Dordr).

[103] Tang T, Yang Z, Zhu Q, Wu Y, Sun K, Alahdal M, et al. Up-regulation of miR-210 induced by a hypoxic microenvironment promotes breast cancer stem cells metastasis, proliferation, and self-renewal by targeting E-cadherin. Faseb j. 2018:fj201801013R. 10.1096/fj.201801013R 30188754

[104] Kelley RK, Gane E, Assenat E, Siebler J, Galle PR, Merle P (2019). A Phase 2 Study of Galunisertib (TGF-β1 Receptor Type I Inhibitor) and Sorafenib in Patients With Advanced Hepatocellular Carcinoma. Clin Transl Gastroenterol.

[105] Neuzillet C, Tijeras-Raballand A, Cohen R, Cros J, Faivre S, Raymond E (2015). Targeting the TGFβ pathway for cancer therapy. Pharmacol Ther.

[106] Colak S, Ten Dijke P (2017). Targeting TGF-β Signaling in Cancer. Trends Cancer.

[107] Zhao S, Mi Y, Guan B, Zheng B, Wei P, Gu Y (2020). Tumor-derived exosomal miR-934 induces macrophage M2 polarization to promote liver metastasis of colorectal cancer. J Hematol Oncol.

[108] Lin X, Wang S, Sun M, Zhang C, Wei C, Yang C (2019). miR-195-5p/NOTCH2-mediated EMT modulates IL-4 secretion in colorectal cancer to affect M2-like TAM polarization. J Hematol Oncol.

[109] Kaschula CH, Tuveri R, Ngarande E, Dzobo K, Barnett C, Kusza DA (2019). The garlic compound ajoene covalently binds vimentin, disrupts the vimentin network and exerts anti-metastatic activity in cancer cells. BMC Cancer.

[110] Lou L, Yu Z, Wang Y, Wang S, Zhao Y (2018). c-Src inhibitor selectively inhibits triple-negative breast cancer overexpressed Vimentin in vitro and in vivo. Cancer Sci.

[111] Gjerdrum C, Tiron C, Høiby T, Stefansson I, Haugen H, Sandal T (2010). Axl is an essential epithelial-to-mesenchymal transition-induced regulator of breast cancer metastasis and patient survival. Proc Natl Acad Sci U S A.

[112] Sheridan C (2013). First Axl inhibitor enters clinical trials. Nat Biotechnol.

[113] Pattabiraman DR, Bierie B, Kober KI, Thiru P, Krall JA, Zill C (2016). Activation of PKA leads to mesenchymal-to-epithelial transition and loss of tumor-initiating ability. Science.

[114] Wang S, Chen Y, Yu X, Lu Y, Wang H, Wu F (2019). miR-129-5p attenuates cell proliferation and epithelial mesenchymal transition via HMGB1 in gastric cancer. Pathol Res Pract.

[115] Yu Y, Zhao Y, Sun XH, Ge J, Zhang B, Wang X (2015). Down-regulation of miR-129-5p via the Twist1-Snail feedback loop stimulates the epithelial-mesenchymal transition and is associated with poor prognosis in breast cancer. Oncotarget.

[116] Zhou Y, Zheng X, Lu J, Chen W, Li X, Zhao L (2018). Ginsenoside 20(S)-Rg3 Inhibits the Warburg Effect Via Modulating DNMT3A/ MiR-532-3p/HK2 Pathway in Ovarian Cancer Cells. Cell Physiol Biochem.

[117] Han J, Wang F, Lan Y, Wang J, Nie C, Liang Y (2019). KIFC1 regulated by miR-532-3p promotes epithelial-to-mesenchymal transition and metastasis of hepatocellular carcinoma via gankyrin/AKT signaling. Oncogene.

[118] Luan QX, Zhang BG, Li XJ, Guo MY (2016). MiR-129-5p is downregulated in breast cancer cells partly due to promoter H3K27m3 modification and regulates epithelial-mesenchymal transition and multi-drug resistance. Eur Rev Med Pharmacol Sci.

[119] Gu C, Cai J, Xu Z, Zhou S, Ye L, Yan Q (2019). MiR-532-3p suppresses colorectal cancer progression by disrupting the ETS1/TGM2 axis-mediated Wnt/β-catenin signaling. Cell Death Dis.

[120] Ning X, Wang C, Zhang M, Wang K (2019). Ectopic Expression of miR-147 Inhibits Stem Cell Marker and Epithelial-Mesenchymal Transition (EMT)-Related Protein Expression in Colon Cancer Cells. Oncol Res.

[121] Chen JH, Huang WC, Bamodu OA, Chang PM, Chao TY, Huang TH (2019). Monospecific antibody targeting of CDH11 inhibits epithelial-to-mesenchymal transition and represses cancer stem cell-like phenotype by up-regulating miR-335 in metastatic breast cancer, in vitro and in vivo. BMC Cancer.

[122] Wang J, Li M, Han X, Wang H, Wang X, Ma G (2020). MiR-1976 knockdown promotes epithelial-mesenchymal transition and cancer stem cell properties inducing triple-negative breast cancer metastasis. Cell Death Dis.

[123] Han S, Shi Y, Sun L, Liu Z, Song T, Liu Q (2019). MiR-4319 induced an inhibition of epithelial-mesenchymal transition and prevented cancer stemness of HCC through targeting FOXQ1. Int J Biol Sci.

[124] Amankwatia EB, Chakravarty P, Carey FA, Weidlich S, Steele RJ, Munro AJ (2015). MicroRNA-224 is associated with colorectal cancer progression and response to 5-fluorouracil-based chemotherapy by KRAS-dependent and -independent mechanisms. Br J Cancer.

[125] Ban E, Kwon TH, Kim A (2019). Delivery of therapeutic miRNA using polymer-based formulation. Drug Deliv Transl Res.

[126] Seok H, Lee H, Jang ES, Chi SW (2018). Evaluation and control of miRNA-like off-target repression for RNA interference. Cell Mol Life Sci.

[127] Yang YP, Chien Y, Chiou GY, Cherng JY, Wang ML, Lo WL (2012). Inhibition of cancer stem cell-like properties and reduced chemoradioresistance of glioblastoma using microRNA145 with cationic polyurethane-short branch PEI. Biomaterials.

[128] Li Y, Zheng Q, Bao C, Li S, Guo W, Zhao J (2015). Circular RNA is enriched and stable in exosomes: a promising biomarker for cancer diagnosis. Cell Res.

[129] Nievergall E, Ramshaw HS, Yong AS, Biondo M, Busfield SJ, Vairo G (2014). Monoclonal antibody targeting of IL-3 receptor α with CSL362 effectively depletes CML progenitor and stem cells. Blood.

[130] Bellavia D, Raimondo S, Calabrese G, Forte S, Cristaldi M, Patinella A (2017). Interleukin 3- receptor targeted exosomes inhibit in vitro and in vivo Chronic Myelogenous Leukemia cell growth. Theranostics.

